# VersorgungsNOT – Psychotherapie als zentrale, aber marginalisierte Versorgungsleistung im Gesundheitssystem: Der Preis der Ignoranz – was kostet es uns als Gesellschaft?

**DOI:** 10.1007/s00729-022-00210-y

**Published:** 2022-12-05

**Authors:** Gabriele Rieß, Henriette Löffler-Stastka

**Affiliations:** 1 Koordinationsstelle Psychotherapieforschung, GÖG, Wien, Österreich; 2 Klinik für Psychoanalyse und Psychotherapie, Medizinische Universität Wien, Wien, Österreich

**Keywords:** Psychotherapieversorgung, Bedarf, Gesellschaft Soziale Verantwortung, Strukturplanung, Psychotherapy care, Need, Society, Social responsibility, Structure planning

## Abstract

Der Artikel geht der gesellschaftsrelevanten Frage nach, welche Versorgungskonzepte und Qualitätssicherungsmaßnahmen erforderlich sind, um der Psychotherapie als Behandlung einen versorgungsrelevanten Stellenwert in der österreichischen Gesundheitsversorgung einzuräumen. Er geht auf soziologische Ursachen für den Anstieg psychischer Krankheiten sowie auf Fakten zum Bedarf, auf Prävalenzzahlen, Versorgungsdaten und -mittel ein und beschreibt, wie die Entwicklung einer Nationalen Strategie Psychische Gesundheit helfen kann, die flächendeckende Versorgung mit Psychotherapie zu verbessern.

Der Artikel beruht auf den Erkenntnissen der Autorinnen aus der Tagung 2020 „VersorgungsNOTwendigkeit – Versorgung in der NOT“ – veranstaltet von der Koordinationsstelle Psychotherapieforschung an der Gesundheit Österreich GmbH – und der vertieften Auseinandersetzung zum Thema Psychotherapieversorgung bzw. Versorgungsnot: In Fokusgruppen bzw. im Fachbeirat der Koordinationsstelle wurden die Fragen der Psychotherapieversorgung diskutiert sowie eine Literaturanalyse und vertiefende exemplarische explorative Interviews zum Thema durchgeführt. Der Artikel bietet erste Ergebnisse dieser Analysen, Recherchen, Reflexionen und die Schlussfolgerungen der Autorinnen.

## Abstract (en)

The article addresses the socially relevant question of which care concepts and quality assurance measures are necessary to give psychotherapy a care-relevant status in the Austrian health care system, also addresses sociological causes for the increase in mental illnesses as well as facts about need, prevalence, health care data and resources, and describes how the development of a National Mental Health Strategy can help to improve the nationwide provision of psychotherapy.

The article is based on the authors’ findings from the 2020 conference “VersorgungsNOTwendigkeit—Versorgung in der NOT” – organized by the Coordination Office for Psychotherapy Research at Gesundheit Österreich GmbH – and the in-depth discussion on the topic of psychotherapy supply or supply shortage: the issues of psychotherapy supply were discussed in focus groups and in the advisory board of the Coordination Office, and a literature review and in-depth explorative interviews on the topic were conducted. The article presents first results of these analyses, searches, reflections and the authors’ conclusions.

## Psychotherapie: marginalisierte Versorgungsleistung im österreichischen Gesundheitssystem – die Pandemie als Chance?

Momentan ist Psychotherapie – 30 Jahre nach Inkrafttreten des Psychotherapiegesetzes – immer noch als Leistung der Sozialversicherung kaum zugänglich, da der Versorgungsausbau nicht im erforderlichen und dem im Sinne der *gesundheitlichen Chancengerechtigkeit* notwendigen Ausmaß erfolgte (Arrouas und Fleischmann 2019). Psychotherapie können sich häufig nur jene Bevölkerungsschichten leisten, die über ein überdurchschnittliches Einkommen verfügen, also eher abgesichert im (Arbeits‑)Leben stehen und in vielen Fällen nicht zu den schwer und schwerst Erkrankten gehören, die diese Versorgungsleistung dringend benötigen würden. Und dies, obwohl die *Prävalenzzahlen psychischer Erkrankungen hoch* sind (GBD 2019 Mental Disorders Collaborators 2022).

Psychotherapie muss im österreichischen Gesundheitssystem langfristig – parallel zur und integriert mit der somatisch-medizinischen Versorgung – jenen Stellenwert erhalten, der ihrem Nutzen entspricht. Um die aus Sicht der Finanzierungsvolumina marginalisierte Position, die der Psychotherapie im Gesundheitssystem eingeräumt wird, zu verändern, sind verschiedene Schritte erforderlich, welche im vorliegenden Artikel resümierend dargestellt werden.

Die Corona-Pandemie bzw. ihre Bekämpfung hatten massive Folgen für die psychische Gesundheit – v. a. bei Kindern und Jugendlichen (Pietrabissa et al. 2021) und machten gleichzeitig deutlich, dass psychische Gesundheit einen zentralen, unerlässlichen Faktor einer gesunden Bevölkerung darstellt. Das durch die Pandemie erstarkte gesellschaftliche Bewusstsein (Riess et al. 2021) für psychisches Leid und somit für Psychotherapie könnte ein förderlicher Nährboden und Ausgangspunkt sein für die konsolidierte Etablierung dieser Versorgungsleistung im Sinne einer Orientierung am *individuellen Bedarf* und *subjektiven Erleben* jener Menschen, die an psychischen Erkrankungen leiden.

## Psychotherapie und gesellschaftlicher Kontext: Demokratie und Angst

Psychische Belastungen/Krankheiten nehmen – nicht erst seit der Pandemie – zu. Dieser alarmierende Trend steht auch in Wechselwirkung mit dem sich seit den 50er-Jahren abzeichnenden und in den letzten 20 bis 30 Jahren deutlicher gewordenen gesellschaftlichen Wandel bzw. mit der funktionellen Ausdifferenzierung moderner Gesellschaften (Löffler-Stastka und Krajic 2022): Ein sozialer Wandel hin zu demokratischeren Strukturen, neue flexible Arbeitszeitmodelle, Leistungsdruck u. v. m. führen zwar oft zu mehr Freiheit und Eigenverantwortung, aber bei manchen Menschen zu mehr Angst und Überforderung und begünstigen somit psychische Erkrankung.

Dieser Wandel kann auch im Bereich der Krankenbehandlung z. B. durch eine Neudefinition des Arzt/Ärztin-Patient:in-Verhältnisses beschrieben werden. Perspektiven von nicht ärztlichen Expert:innen bzw. ökonomische Überlegungen drangen in den Hoheitsbereich der Krankenbehandlung ein, womit auch Regulierungsprozesse miteinhergingen (vgl. Einführung der Leistungsbezogenen Krankenhausfinanzierung/LKF 1995, des Psychotherapiegesetztes 1991, des Unterbringungsgesetzes 1990, etc.): dies führte zur Ökonomisierung, Technisierung, Regulierung bzw. „Verrechtlichung“ der Medizin, aber eben auch zu wichtigen Demokratisierungsprozessen.

Andererseits sind liberale Gesellschaften wegen fehlender stützender Orientierung oder Ungleichheiten im Bildungszugang immer weniger geschützt gegenüber der zunehmenden Verbreitung von Fake News und paranoiden Exzessen, wodurch *gesellschaftliche Spaltungsprozesse* befördert werden, die ihrerseits mehr Integration und Solidarität nötig machen, um der gesellschaftlichen Spaltung entgegenzuhalten. In diesem Spannungsfeld kommt der Psychotherapie ein wichtiges Gestaltungsmoment zu. Psychotherapeut:innen können auf Ebene der Gesundheitsversorgung eine wichtige Rolle einnehmen: Auf Basis einer tragfesten Beziehung fördern sie sowohl Autonomie als auch Angstreduktion und (Selbst‑)Reflexion verbunden mit (Selbst‑)Verantwortung im Sinne der psychischen Gesundheit. Dies wiederum ist Basis für demokratische, rationale Meinungsbildung und gesellschaftliche Teilhabe. Gesellschaftlichen Spaltungsprozessen kann so ein stabilisierendes (Selbst‑)Vertrauen entgegengehalten werden.

Wie aber stellt sich die Ausgangslage (bzgl. Prävalenz psychischer Erkrankungen und Psychotherapiebedarf) für die Versorgung mit Psychotherapie dar und welchen ökonomischen Vorteil hätte eine Gesellschaft von einer funktionierenden Versorgung?

## Prävalenz, Psychotherapie-Bedarf und ökonomische Bedeutung psychischer Erkrankungen

Die OECD und die Europäische Kommission beziffern die *(Folge‑)Kosten psychischer Erkrankungen* in Österreich für das Jahr 2019 mit rund 4,3 % des Bruttoinlandsproduktes auf etwa 13,9 Mrd. € und betonen neben diesem finanziellen volkswirtschaftlichen Schaden auch extrem hohe individuelle sowie familiäre seelische Belastungen (*Global Burden of disease*, vgl. OECD 2020 [2015]; GBD 2019 Mental Disorders Collaborators 2022; Rechnungshof 2019; Nübling et al. 2014; Statistik Austria 2020; Wancata 2017). Psychische Erkrankungen wie Angst, affektive Störungen inkl. Depression oder somatische Belastungsstörungen haben die somatischen Erkrankungen im Ranking der häufigsten Krankheitsbilder in Österreich mittlerweile überholt (vgl. auch Löffler-Stastka und Hochgerner 2021). Dieser Trend wird aufgrund der COVID-19-Folgeerkrankungen um weitere 20 % steigen (Pietrabissa et al. 2021).

Eine Befragung zu Prävalenz und Versorgung psychischer Krankheiten in Österreich (Wancata 2017) an einer österreichweit repräsentativen Stichprobe ergab eine 1‑Jahres-Prävalenz von rund 23 % psychischer Symptome und Erkrankungen (erhoben mittels der „Present State Examination PSE-10“ der „Schedules for Clinical Assessment in Neuropsychiatry SCAN“ zur psychiatrischen Fallidentifikation). Bei der Untersuchung des Bedarfs zeigte sich, dass nach klinischer Einschätzung der Untersucher:innen rund 14 % der Bevölkerung mit Psychotherapie versorgt werden sollten (Wancata 2017; vgl. auch Löffler-Stastka und Hochgerner 2021).

## Hoher Nutzen von Psychotherapie in ökonomischen Analysen

Mit Psychotherapieversorgung befasste Gesundheitsökonom:innen konstatieren – im Kontrast zur Versorgungslage – eine *eindrückliche Kosten-Nutzen-Effizienz für Psychotherapie* im Vergleich zu anderen (rein somatischen) Interventionsformen (Rechnungshof 2019; Seitz et al. 2019; Nübling 2014; Margraf 2009). Eine Metaanalyse über 54 verfügbare Studien innerhalb von 10 Jahren mit mehr als 13.000 Patient:innen zeigt in 86 % der analysierten Studien eine Netto-Einsparung durch Psychotherapie (positives Kosten-Nutzen-Verhältnis nach Abzug der Psychotherapiekosten) nach ca. 2 Jahren (zeitlich stabile Kostenreduktionen bei stationären Leistungen und Arbeitsausfallkosten) sowie in 76 % der Studien eine Überlegenheit (bzw. einen signifikanten Zusatznutzen) in der Wirksamkeit gegenüber medikamentösen Strategien (Margraf 2009). Ebenso zeigen Kosten-Nutzen-Analysen zu Psychotherapie die Effizienz im Vergleich zu den hohen Folgekosten psychischer Erkrankungen. Beispielsweise konnten die Kosten (inkl. sekundäre Kosten wie Krankenstandstage, etc.) bei Patient:innen mit chronischer somatischer Belastungsstörung durch die in Frequenz und Dosis adäquate, integrierte psychotherapeutische Behandlung auf durchschnittlich € 80.000,– pro Patient:in pro Jahr halbiert werden (Seitz et al. 2019).

Psychotherapie wäre also nicht nur *wirksamer im Vergleich zu medikamentösen Strategien*, sondern langfristig auch *billiger als keine Therapie *(Nettoeinsparung durch Psychotherapie nach ca. 2 Jahren vgl. Margraf 2009), würde sie denn solidarisch – von der Sozialversicherung – finanziert werden und im Sinne des Patientenrechts auf Versorgungszugang jenen, die an einer psychischen Erkrankung leiden, auch zur Verfügung stehen.

## Aktuelle Versorgungssituation in Österreich

Die Versorgung mit Psychotherapie stellt in Österreich eine gewisse Basisversorgung dar. Die Ausgaben für Psychotherapie wurden in den letzten Jahren aber nur rudimentär aufgestockt. Derzeit werden (bezogen auf die Zahlen aus 2014 in Grabenhofer-Eggerth et al. 2020) rund 237.000 Patient:innen mit psychotherapeutischen Leistungen versorgt, also 2,8 % der Gesamtbevölkerung. Davon wurden über Versorgungsvereine und Institutionen etwas über 60.000 Personen (25 %) erreicht, bezuschusste Psychotherapie erhielten rund 56.000 (34 %) und 3700 Personen (2 %) wurden in kasseneigenen Einrichtungen versorgt. Durch ärztliche psychotherapeutische Leistungen wurden rund 117.000 Personen (49 %) versorgt (ebd. 2020).

Von der solidarischen Gesundheitsversorgung werden pro Jahr rund 76,4 Mio. € (bezogen auf die Zahlen aus 2014 in Grabenhofer-Eggerth et al. 2020) für die psychotherapeutische Versorgung aufgebracht. Von den Gesamtausgaben entfielen 60 % auf durch Versorgungsvereine und Institutionen erbrachte Psychotherapie, 20 % auf Leistungen durch Vertrags- und Wahlärzte, 19 % auf Kostenzuschuss zur psychotherapeutischen Behandlung bei niedergelassenen Psychotherapeut:innen und 1 % auf Leistungen kasseneigener Einrichtungen. Die Ausgaben pro versicherte Person *variieren regional beträchtlich* und orientieren sich nicht an einheitlichen Bedarfswerten. Außerdem unterscheiden sich Leistungen der Vertrags‑/Wahlärzte meist von der in Versorgungsvereinen und Institutionen erbrachter Psychotherapie, zumal die Dauer ihrer Leistungen – ähnlich wie auch in kasseneigenen Einrichtungen – kürzer als 50 min ist und sich auf wenige (2–3) Sitzungen beläuft.

Dies zeigt, dass im Versorgungssystem aus Sicht der Sozialversicherungsträger *uneinheitlich definiert* ist, was unter Psychotherapie verstanden wird bzw. die (klassische) Psychotherapie durch Psychotherapeut:innen mit einem vergleichsweise geringen Finanzvolumen ausgestattet ist.

Studien für Österreich (Riess et al. 2021) zeigen weiters, dass ein niederschwelliger, kostenfreier *Zugang* zu Psychotherapie dringend erforderlich und die gesetzliche Grundlage dafür seit langem geschaffen ist (Springer-Kremser et al. 2002). Die Antrags- und Bewilligungspraxis der Krankenkassen ist aber kompliziert, was eine Hürde im Versorgungszugang und eine hohe Belastung für Patient:innen bedeutet.

Die Versorgungslage im öffentlichen Gesundheitssystem ist für Psychotherapie aber keineswegs so, wie sie für jene Menschen, die Psychotherapie dringend benötigen würden, sein sollte oder wie es Kosten-Nutzen-Analysen nahelegen. Wieso?

## Die Rolle des gesellschaftlich-kollektiven Bewusstseins und die Fallstricke in der Diskussion zur Psychotherapieversorgung

*Psyche* und *Psychische Gesundheit* sind als Begriffe und systematisches Wissen noch immer *völlig unterrepräsentiert *in der Gesellschaft/im kollektiven Bewusstsein, v. a. aber – und das ist für das gegenständliche Thema entscheidend – auch *im Gesundheitssystem*. Wenngleich die Corona-Pandemie zumindest das kollektive Bewusstsein für psychisches Leid erhöht hat, wird psychische Gesundheit im Gesundheitssystem meist unsystematisch thematisiert, denn es fehlt häufig ein *kohärentes (theoretisches) Verständnis* (bzw. eine *genaue Definition*) von Psyche und psychischer Gesundheit/Krankheit sowie ihrer Förderung/Behandlung durch Psychotherapie (Watzka 2022; Löffler-Stastka et al. 2009).

Folglich werden oft nur *Ausschnitte und Versorgungsfragmente* diskutiert, welche dann mit überproportionaler Energie und Finanzmittel ausgestattet werden. Es geht dann entweder ausschließlich um ein spezifisches Setting (z. B. intramural, weniger extramural/ambulant/niedergelassen), eine bestimmte Zielgruppe (z. B. Altersgruppe wie Kinder/Jugendliche), eine einzelne Behandlungsphase (z. B. Krankenbehandlung bzw. Prävention) oder ein besonderes Störungsbild (z. B. Abhängigkeitserkrankungen). Die breite Palette *aller* psychischen Erkrankungen (z. B. auch Angsterkrankungen, affektive Störungen inkl. Depressionen oder somatische Belastungsstörungen) bzw. aus Prävalenzsicht dringliche Erkrankungen spielen in der öffentlichen Thematisierung sowie in der Versorgungsdiskussion kaum eine Rolle.

Ein weiterer Fallstrick der Diskussion ist, dass *Fragen der Fachdisziplin Psychotherapie* selten als integriertes Gesamtpaket dieser Berufsgruppe (unter Inklusion angrenzender Fachdisziplinen) zugeordnet und mit dieser gemeinsam diskutiert werden: Es ist ein im Gesundheits‑/Sozialsystem häufig ausgeblendetes Faktum, dass die psychotherapeutische Arbeit aus *unterschiedlichen Handlungsfeldern* und *Angeboten in verschiedenen Settings* mit *unterschiedlicher Frequenz und Behandlungsdauer* besteht. Dabei sind die *Behandlungs- bzw. Arbeitsphasen* der Psychotherapie von durchaus *sehr differenzierten Zielen* geleitet, welche wiederum ineinandergreifen: z. B. Heilung, Linderung, Stabilisierung bzw. Vermeidung der Krankheitsverschlechterung, weiters Prävention und psychosoziale Beratung etc. in unterschiedlichen Settings. Werden diese *unterschiedlichen psychotherapeutischen Handlungsfelder* inkl. ihrer Ziele getrennt voneinander diskutiert, verlangsamen Berufsgruppenrivalitäten bzw. Kompetenzstreitigkeiten (vor allem an den fachlichen Schnittstellen) die notwendige Versorgungsverbesserung, verstärken die Fragmentierung des Gesundheitssystems und erhöhen jedenfalls die Unübersichtlichkeit (bzgl. Institutionen und Berufsgruppen) für Patient:innen bzw. Hilfesuchende.

Auch die Diskussion von Ausbildungsdetails oder zu Qualitätsüberprüfung von Psychotherapie-/verfahren (als ginge es um ein neues Behandlungsverfahren im Gesundheitsdiskurs) sowie das Hereinholen neuer Berufsgruppen in das ASVG als *Kompensation für die vorliegende Mangelversorgung* mit Psychotherapie lösen folglich das Problem nicht. Die *Vermischung* bzw. das *Ausblenden* der Versorgungsebenen führt in Folge zur wechselseitigen Delegation von Finanzierungsverantwortung (vgl. Rechnungshofbericht 2019), wodurch letztlich ein Großteil der psychotherapeutischen Versorgung auf den Privatsektor (Zahlung „out of pocket“) verschoben wird. Das Problem der Unterversorgung kann NICHT allein durch eine *Steuerung des Zugangs und „sparsame“ Ressourcenallokation* (Stichwort: Mangelverwaltung) behoben werden.

## Gegenstandsbereich von Psychotherapie und Subjektbegriff: Fachdisziplin und intuitives Alltagswissen

Möglicherweise erschwert ein spezifischer Umstand die Diskussion und trägt insbesondere zu den *auffälligen Zuständigkeitsstreitigkeiten* des psychosozialen Felds bei: Der Vor- und Nachteil des Kompetenz- und Berufsprofils der Psychotherapie ist seine am (in eine kollektive Matrix eingebetteten) Individuum und dessen subjektiv-persönlichen Erlebensweisen ausgerichtete Behandlungsform (Riess 2018). Diese hat die *Autonomieentwicklung und Selbstregulation des Menschen* im Auge und steht somit der klassischen Vorstellung einer somatisch-medizinischen Behandlung entlang biologisch-physikalisch-chemischer Paradigmen und Parameter scheinbar entgegen. Psychotherapie kann gemäß ihrem Selbstverständnis nichts außer Acht lassen, was im *subjektiv bewussten, existentiellen, vor- und unbewussten Erleben und Körpererleben* eines sozial-historisch-gesellschaftlich und ökonomisch eingebetteten Menschen wirksam und von Bedeutung ist. Selbstverständlich haben auch die Medizin und alle gesundheitsrelevanten Berufe im medizinischen Gesundheitssystem sowie im Sozialsystem (v. a. Sozialarbeit) mit diesen subjektiven und psychologischen Aspekten des Mensch-Seins zu tun und tangieren sie in ihren Arbeits- oder Behandlungsformen; aber die Psychotherapie ist jene (sehr fach-interdisziplinär arbeitende) Disziplin (Wampold und Imel 2015), die sich auf diese Fragestellung der Psychischen Gesundheit und Krankheit bzw. ihrer Gegenstandsdefinition – basierend auf den psychologischen Diskursen zu Erleben und Verhalten des Menschen – sowie ihre Weiterentwicklung und Behandlung (inkl. gegenstandsspezifischer Präventionsarbeit) als *Fachdisziplin* konzentriert.

## Mangelversorgung durch Unterfinanzierung oder Verbesserung der Psychotherapieversorgung?

Das *Ausbleiben der Budgetaufstockung* (z. B. durch die Sozialversicherung) muss folglich stärker für die Mangelversorgung verantwortlich gemacht werden als etwa die Komplexität des Gegenstandsbereiches Psychotherapie bzw. eine mangelnde „Integration“ der Versorgung: Eine nicht existente Versorgungsstruktur „Psychotherapie“ kann auch nicht mit anderen Berufsgruppen bzw. mit dem medizinischen oder sozialen Sektor „integriert“ angeboten werden. Ohne den Aufbau einer systematischen Psychotherapieversorgung durch die entsprechende *Verantwortung für die Finanzierung* kann es auch keine wie auch immer geartete „integrierte Versorgung“ (vgl. Klein et al. 2012) geben und setzt sich die Stigmatisierung psychisch Erkrankter fort.

Als (erste) Schlussfolgerungen der beschriebenen Analysen (vgl. Tagung 2020 und vertiefte Auseinandersetzung in Fokusgruppe bzw. Fachbeirat, explorative Interviews und Literaturrecherche) wurden aus Sicht der Autorinnen mehrere qualitätssichernde Maßnahmen als relevant und dringlich abgeleitet, welche im Sinne einer aktuellen Nationalen Strategie Psychische Gesundheit konzipiert und bearbeitet werden könnten.

### Versorgungsgerechtigkeit und bedarfsgerechte Versorgungsstruktur mit entsprechendem Budget

Wichtiges Prinzip in Bezug auf die Verbesserung der psychotherapeutischen Versorgungslage *im Sinne einer Orientierung am individuellen Bedarf und subjektiven Erleben/Leid von Betroffenen* ist, dass zunächst für Versorgungsgerechtigkeit gesorgt wird, und zwar durch die *Bereitstellung einer bedarfsgerechten Versorgungsstruktur Psychotherapie mit entsprechendem Budget*, die sich an der international gut untersuchten Prävalenz psychischer Erkrankungen (WHO, Deutschland, Schweiz) orientiert bzw. an den Bedarfsraten von Hilfebedürftigen, die an das System der Psychotherapieversorgung angebunden werden können (Löffler-Stastka und Hochgerner 2021).

### Qualitätssicherungsinstrument Versorgungsdaten, Survey Psychische Gesundheit und Angebotserfassung

Da die Datenlage zur Psychotherapie(versorgung) in Österreich lückenhaft ist, ist es erforderlich, eine flächendeckende Gesundheitsberichterstattung Psychotherapie (Dokumentationssystem Psychotherapie PsyDok) zu konzipieren und aufzubauen. Diese sollte ergänzt werden durch die Erarbeitung eines Konzepts für einen nationalen Gesundheitssurvey Psychische Gesundheit (PsyG-Dok, vgl. Gesundheitssurvey Deutschland), welcher typische (klinische) Screening-Instrumente verwendet und eine wichtige Grundlage zu aktuellen österreichischen Prävalenzdaten als Basis für eine *bedarfsgerechte Angebotsplanung* liefern würde (vgl. Rechnungshof 2019).

Die Erarbeitung eines mehrstufigen Budgetplans, d. h. eine verbindliche *Finanzierungsstruktur/Finanzierungszuständigkeit und Budgethöhe *für Psychotherapie in allen Settings, insbesondere jedoch im niederschwelligen ambulanten/niedergelassenen Setting (Psychotherapie-Ambulanzen und niedergelassene Psychotherapie) wäre zweckmäßig.

Die Erfassung (mittels einer zu konzipierenden Erhebung) und Analyse der Strukturqualität *aller vorhandenen Angebote* im psychosozialen Feld (psychosoziale Institutionen) bzw. jener Angebote, die den stationären mit dem extramuralen Versorgungsbereich verknüpfen (vgl. Entlassungsmanagement), wäre ein wichtiger Schritt, um Übersicht über relevante Systempartner der Psychotherapie zu erhalten. Ein solches Erhebungskonzept sollte das Stundenausmaß der Beschäftigten, ihrer Berufe bzw. Weiterbildungen sowie ihre Kompetenz- und Aufgabenprofile inkludieren. Die Strukturanalyse muss auch die Finanzierungstruktur erfassen.

### Strukturplanung für Psychotherapie-Ambulanzen und niedergelassene Psychotherapie

Auf Basis der Ergebnisse oben vorgeschlagener Erhebung sollte für den ambulanten Psychotherapieversorgungbereich eine einheitliche, idealtypische Struktur für *Psychotherapie-Ambulanzen* (Struktur- inkl. Prozessplanung) festgelegt (vgl. Child Guidance Clinic, ÖAGG psychotherapeutische Ambulanz gGmbH, div. psychotherapeutische (Universitäts‑)Ambulanzen) und mittel- bis langfristig im Österreichischen Strukturplan Gesundheit ÖSG (Eglau et al. 2021) umsetzungswirksam verankert werden.

### Differenzierte Bedarfs- und Angebotsplanung u. a. in der niedergelassenen Psychotherapie

Die Definition *differenzierter Bedarfs- und Angebotsformen* insbesondere für das ambulante Setting der *niederschwelligen* niedergelassenen Psychotherapie-Praxen ist insofern wichtig, als dadurch der breite Begriff Psychotherapie für Verantwortliche im Gesundheitssystem verständlich aufgeschlüsselt und sichtbar gemacht wird. Völlig unabhängig von der methoden-/schulenspezifischen Ausrichtung eines Psychotherapieverfahrens muss ein/e Psychotherapeut:in je nach *individuellem Bedarf* bzw. Problem- und Störungslage der Patient:innen entscheiden, welches Angebot er/sie setzt. Außerdem muss die Definition der Versorgungswirksamkeit im Verhältnis zum Zeitaufwand (der Praxisführung) festgelegt werden.

### Psychotherapieplanung: Psychotherapeutische Diagnostik und Indikationsstellung

Im Zuge der *psychotherapeutischen Diagnostik* muss eine *differenzierte i.e. individuelle Psychotherapieplanung* mit dem Ziel der *psychotherapeutischen Indikationsstellung* erfolgen: Sie unterscheidet beispielsweise zwischen (1) Krisenintervention/Akutbehandlung, (2) psychosozialer Beratung und (3) Psychotherapie, welche als (3a) Kurz- oder (3b) Langzeitpsychotherapie nieder- und hochfrequent geplant werden kann und im Zuge der probatorischen Sitzungen erprobt werden sollte.

Die fachliche Definition und Klärung der Kriterien für die differenzierte psychotherapeutische Indikationsstellung zu spezifischen Bedarfen bzw. Angeboten muss mittels Fachexpertise und spezifischer Diagnostik-Instrumente (z. B. Operationalisierte Psychodynamische Diagnostik) erfolgen, um Kriterien für schwer Erkrankte, aber natürlich auch für andere behandlungsbedürftige Patient:innen- und Diagnosegruppen zu definieren und entsprechenden Angeboten zuordnen zu können. Dabei müssen weitere fachliche Kriterien – u. a. die aktuelle *Psychotherapiefähigkeit* – berücksichtigt werden. Entsprechende Empfehlungen können in Anlehnung an bereits etablierte Vorgehensweisen, wie sie etwa im Tiroler- oder Salzburger Modell praktiziert werden, formuliert werden.

### Differenzierte Angebotsformen der Psychotherapie und definierte Stundenkontingente

Für all diese *am individuellen Bedarf orientierten differenzierten Angebotsformen* sollten *jährliche Stundenkontingente* aus Fachsicht definiert werden, die beispielsweise ein Stundenausmaß von 1–10 h für das Angebot Psychotherapeutische Diagnostik, Indikationsstellung & Therapieplanung bzw. Krisenintervention/Akutpsychotherapie oder psychosoziale Beratung (vgl. Lambert 2013) bzw. ein Stundenausmaß von 10–25 h für das Angebot Psychotherapie als Kurzbehandlung (bzw. Fokaltherapie) bzw. 25–40 (ggf. 80–160 bei höherfrequenten Therapien) Stunden pro Jahr für das Angebot der Langzeitbehandlung umfassen. Für die Langzeitbehandlung muss darüber hinaus auch die *maximale Zeitspanne* (in Jahren) für Psychotherapie im Rahmen der Versicherungsleistung transparent diskutiert und geplant werden.

### Die Patient:innenperspektive

Die allgemeine Natur der Psychotherapie spiegelt einen *intimen, sehr persönlichen und an individuellen Bedürfnissen und Bestrebungen bzw. an der subjektiven Erlebnisweise orientierten zwischenmenschlichen Prozess* wider, der im genuinen Interesse bzw. zum persönlichen Nutzen der Patien:in/Klient:in durchgeführt wird: diese Orientierung an der *Perspektive der Hilfesuchenden* fehlt oft in der Diskussion um Gesundheitsversorgung und muss gerade in der psychotherapeutischen Versorgungsplanung (differenzierte Bedarfsorientierung mit entsprechenden differenzierten Angebotsformen) abgebildet sein.

### Niederschwelliger Zugang ins Hilfssystem: die Psychotherapeutische Sprechstunde

Im Sinne eines *niederschwelligen* und *extrem erleichterten Zugangs* könnte für die Leistungsschritte Psychotherapeutische Diagnostik, Indikationsstellung und Festlegen von Art der Intervention bzw. Krisenintervention/Akutpsychotherapie eine Art „Psychotherapeutische Sprechstunde“ in niedergelassenen Psychotherapeutischen Praxen konzipiert und eingeführt werden, wodurch anfragenden Patient:innen z. B. innerhalb von 14 Tagen der erste Termin von bspw. 2–10 *antrags- und kostenfreien Stunden* zur Verfügung gestellt werden kann. Innerhalb dieser Erstgespräche sollte entweder die Akut- oder Krisenintervention und/oder eine psychotherapeutische Diagnostik und -Indikationsstellung zur weiteren (differenzierten) Therapieplanung, eine Kooperation mit Nachbardisziplinen oder eine psychosoziale Beratung bzw. Weitervermittlung ins psychosoziale Feld erfolgen – im Sinne einer *geschlossenen Versorgungskette*.

Qualitätssicherungsinstrumente für die Kooperation (Stichwort: Fall- und Helferkonferenzen) und das *Nahtstellenmanagement* zu benachbarten Berufsgruppen und Angeboten im psychosozialen Feld (insbesondere zu Sozialarbeit und Klinischer Psychologie) sollten erarbeitet bzw. vereinheitlicht zur Verfügung gestellt werden.

### Definition der Psychotherapeutischen Kompetenz: Berufsprofil und Curriculum

Die Ausarbeitung des konkreten *Berufs- und Kompetenzprofils Psychotherapie* (als Förderung der Berufsgruppenentwicklung), wie es für andere Gesundheitsberufe schon lange selbstverständlich ist (vgl. Clementi et al. 2004) sowie die Definition von zu erreichenden Ausbildungszielen eines (akademischen) Curriculums Psychotherapie wären zielführend. Dies kann auch Vergleichsgrundlage zu Kompetenzprofilen der Nachbardisziplinen sein.

### Psychotherapiewissenschaftliches Verständnis von Gesundheit und Krankheit der Psyche

Als Grundlage für die Strukturplanung von Psychotherapieversorgung muss das *Gesundheits- und Krankheitsverständnis für psychische Gesundheit aus psychotherapiewissenschaftlicher Sicht* formuliert werden (Riess 2018, 2016). Außerdem sind die Bestrebungen der vier cluster-spezifischen Theorie-Praxismodelle bzw. die Formulierung von Kriterien (u. a. konsistentes Gesundheits-Krankheits-Modell), die für diese fachinternen Spezifizierungen notwendig sind, auszuformulieren.

Entscheidend ist, dass die Psychotherapieversorgung zwar vernetzt zu anderen Strukturen der psychosozialen Versorgung gedacht wird, da sie ein Teil davon ist, aber als *eigenständiges Versorgungsziel* konzipiert wird, um nicht jenen Fallstricken aufzusitzen, die oben diskutiert wurden (Strotzka 1983).

## Conclusio

Dem Problem der Unterrepräsentation von Psychischer Gesundheit und den sie fördernden Berufsgruppen bzw. dem Mangel an Psychotherapie kann nur anhand einer übergreifenden, systematischen Strategie, forschungsgeleitet basierend auf einem psychotherapiewissenschaftlichen Verständnis von Psychischer Gesundheit und Krankheit begegnet werden, welche alle Handlungsfelder der Psychotherapie einschließlich ihrer Settings, Störungs- und Zielgruppen sowie alle Kooperations‑/Nahtstellenpartner:innen und Finanzierungsverantwortlichen bzw. Entscheidungsträger in den Blick nimmt, um die Versorgung mit – insbesondere ambulanter – Psychotherapie zu verbessern.

Den vielen Problemstellungen (etwa der Unterversorgung bestimmter Zielgruppen wie Kinder und Jugendliche) oder Behandlungsphasen (wie etwa Prävention) oder Symptomgruppen (z. B. Abhängigkeitserkrankungen) oder gesellschaftlich-soziologischen Problemen (z. B. Stigmatisierung) kann nur dann effektiv begegnet werden, wenn eine wesentliche Versorgungsbasis aufgebaut wird, die Grundvoraussetzung für die Behandlung all dieser Themenfelder ist und seit Jahren u. a. von Gesundheitsökonom:innen gefordert wird: die Versorgung mit Psychotherapie insbesondere im *niederschwelligen* niedergelassenen *Setting*.

Die Gleichstellung von psychischer mit physischer Gesundheit sowie ihrer Wiederherstellung durch Psychotherapie mit somatisch-medizinischer Versorgung ist in der gesetzlichen Gleichstellung der betreffenden Berufsgruppen bereits grundgelegt. Ihre Umsetzung in allen Settings erfordert – parallel und integriert zu allen oben genannten Maßnahmen, die in einer Nationalen Strategie Psychische Gesundheit festgelegt werden sollten, – als dringlichsten nächsten Handlungsschritt die Erstellung eines bedarfsorientierten Struktur- und Budgetplans für die nächsten Jahre zum Ausbau der Psychotherapieversorgung in Österreich, was sich v. a. im ambulanten/niedergelassenen Setting in einer massiven *Erhöhung der Ausgaben* niederschlagen sollte.
